# Essential Kinases and Transcriptional Regulators and Their Roles in Autoimmunity

**DOI:** 10.3390/biom9040145

**Published:** 2019-04-10

**Authors:** Ya Nan Deng, Joseph A. Bellanti, Song Guo Zheng

**Affiliations:** 1 Department of Clinical Immunology, Sun Yat-sen University, Guangzhou 510630, China; dengyn7@mail2.sysu.edu.cn; 2 Departments of Pediatrics and Microbiology-Immunology, Georgetown University Medical Center, Washington, DC 20057, USA; bellantj@georgetown.edu; 3 Department of Internal Medicine, Ohio State University College of Medicine and Wexner Medical Center, Columbus, OH 43201, USA

**Keywords:** autoimmunity, kinases, transcriptional factors

## Abstract

Kinases and transcriptional regulators are fundamental components of cell signaling that are expressed on many types of immune cells which are involved in secretion of cytokines, cell proliferation, differentiation, and apoptosis. Both play important roles in biological responses in health as well as in illnesses such as the autoimmune diseases which comprise at least 80 disorders. These diseases are caused by complex genetic and environmental interactions that lead to a breakage of immunologic tolerance and a disruption of the balance between self-reactive cells and regulatory cells. Kinases or transcriptional regulatory factors often have an abnormal expression in the autoimmune cells that participate in the pathogenesis of autoimmune disease. These abnormally expressed kinases or transcriptional regulators can over-activate the function of self-reactive cells to produce inflammatory cytokines or down-regulate the activity of regulatory cells, thus causing autoimmune diseases. In this review we introduce five kinds of kinase and transcriptional regulator related to autoimmune diseases, namely, members of the Janus kinase (JAK) family (JAK3 and/or tyrosine kinase 2 (TYK2)), fork head box protein 3 (Foxp3), the retinoic acid-related orphan receptor gamma t (RORγt), and T-box expressed in T cells (T-bet) factors. We also provide a mechanistic insight into how these kinases and transcriptional regulators affect the function of the immune cells related to autoimmune diseases, as well as a description of a current drug design targeting these kinases and transcriptional regulators. Understanding their exact role helps offer new therapies for control of the inflammatory responses that could lead to clinical improvement of the autoimmune diseases.

## 1. Introduction

Immune tolerance plays an important physiological role in maintaining the stability of the immune system in the healthy host. However, when, due to certain endogenous and external causes, immune tolerance is broken and continuous immune responses to autoantigen(s) are activated that cause abnormal loss of cells, tissue damage, or dysfunction, the emergence of subclinical autoimmune responses is seen, which can also lead to fully expressed clinical autoimmune disease [1,2]. The over-activation of immune responses directed at auto-antigens, therefore, is a very important seminal cause of autoimmune disease. The initial autoantibody attack to self-tissue is usually followed by an inflammatory response caused by autoreactive cytotoxic cells. Autoimmune diseases comprise at least 80 disorders, including systemic lupus erythematosus (SLE), rheumatoid arthritis (RA), and multiple sclerosis (MS), often caused and controlled by complex genetic and environmental interactions [3]. T-cell mediated damage induced by various antigens is a very important cause of organ damage and is often influenced by cytokines such as interferon (IFN)-γ, interleukin (IL)-17, and IL-4 that are pivotal in the pathogenesis of the autoimmune diseases. 

Kinases and transcriptional regulators play important roles in the function of immune cells [4,5]. These kinases or transcriptional regulators are thought to be the molecular antecedents that can activate the function of self-reactive cells by producing inflammatory factors or by down-regulating the activity of regulatory cells leading to the development of the autoimmune diseases. 

**Figure 1:**
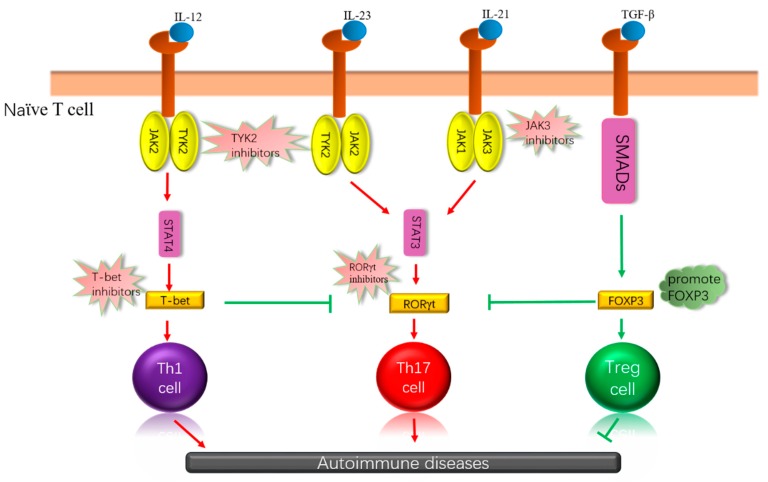
Cytokines are combined with receptors that can influence the differentiation of immune cells through different signaling pathways, which may have different effects on autoimmune disease. Some essential kinases and transcription factors are involved, which, depending on how they work, inhibit, or promote these kinases or transcription factors, can achieve the purpose of treating autoimmune diseases. IL: interleukin, JAK: Janus kinase; TGF-β: transforming growth factor β, SMADs: drosophila mothers against decapentaplegic, STAT: signal transducers and activators of transcription, T-bet: T-box expressed in T cells, RORγt: retinoic acid-related orphan receptor gamma t, Foxp3: forkhead box protein 3.

Currently, there are several clinically available immunomodulatory drugs for the treatment of inflammatory or autoimmune diseases. However, since these drugs have the low discriminative capacity they often cause undesirable side effects [6]. Since several studies have shown that overexpression or deletion of kinases and transcription factors may lead to inflammatory and autoimmune diseases, searching for specific kinases and transcription factors related to autoimmunity and designing clinical therapeutic trials based on their characteristics would appear to be a reasonable strategy for the development of new therapeutic agents with reduced adverse side effects. Therefore, in this review, we mainly introduce several essential kinases and transcription factors involved in autoimmunity, their mechanisms of action, and the current development of drugs that target these kinases and transcription factors (Figure 1). 

## 2. Protein Kinases

**Figure 2:**
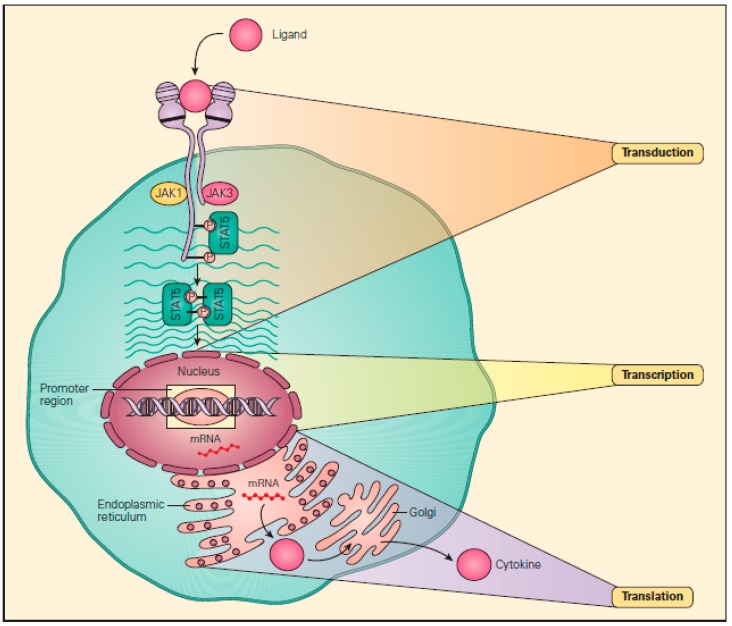
Transduction, transcription, and translation (the “three Ts”). These are the three steps in signaling pathways involved in protein synthesis, beginning with the binding of a ligand with a cell surface receptor initiating the first “T,” transduction, i.e., to lead across. This then proceeds to the phosphorylation of a number of cascading substrate molecules by kinases (i.e., phosphorylases) that results in the production of a transcription factor that binds to a promoter region of DNA within the nucleus. This then initiates the second “T,” transcription, i.e., to write across, during which messenger RNA (mRNA) is formed. After leaving the nucleus, the mRNA sits on a ribosome within the endoplasmic reticulum and acts as a “blueprint” to initiate the third “T,” translation, i.e., to carry across, culminating in the assembly of amino acids into a final protein. (Reproduced with permission from Bellanti, J.A. *Immunology IV: Clinical Applications in Health and Disease*. I Care Press: Bethesda, MD, USA, 2012) [8].

Protein kinases are proteins that phosphorylate serine, threonine, or tyrosine residues of target adaptor proteins. The protein kinases are encoded by 512 kinase genes within the human genome [7] and initiate transduction, the first of the three phases of cell signaling that lead to protein synthesis. These three phases are transduction, transcription, and translation [8] (Figure 2). Kinases are found within the intracellular matrix of T and B lymphocyte surface receptors, and once the receptors bind to extracellular ligands, kinases are activated and initiate intracellular signaling by phosphorylation of a cascading set of adaptor proteins and, once phosphorylated, are referred to as transcription factors that eventually initiate transcription, the second phase of cell signaling [9]. More than 400 diseases are directly or indirectly associated with protein kinases [10]. 

### 2.1. The Janus Kinases Family 

Janus kinases (JAKs) are tyrosine receptor kinases that are associated with many cytokine receptor signaling pathways. Members of the JAKs family include at least four different types of tyrosine kinases (tyrosine kinase 2 (TYK2), JAK1, JAK2, and JAK3), each playing a critical role in many biological responses, such as the secretion of cytokines, cell proliferation, differentiation, and apoptosis [11]. Janus kinases are also capable of activating partner transcription factors such as the signal transducers and activators of transcription (STATs) through trans phosphorylation. Collectively, the JAKs together with STATs regulate the expression of cytokine-induced genes, thus providing a formidable combination that controls a series of physiological processes [12,13]. JAKs are relatively large kinases of about 1150 amino acids and molecular weights of approximately 120–130 kDa. Their mRNA transcription range is 4.4–5.4 Kb. When the cytokines bind to their corresponding receptors, JAKs, they become enzymatically active and acquire the unique capacity to not only phosphorylate their own structures, but also receptor chains and other substrates, including the STATs [14]. Among these structures, the JAK/STAT pathway is an important signaling pathway, and activation of the JAK/STAT pathway permit cytokines to regulate the differentiation, development, and function of T cells and bone marrow cells [15].

#### 2.1.1. Janus Kinase 3

Although JAK1, JAK2, and TYK2 can universally express the function in vivo, JAK3, in contrast, has more restricted tissue expression. It is highly expressed in natural killer (NK) cells and thymocytes and can be induced in T, B, and bone marrow cells. Mutations of JAK3 can lead to loss of function and severe immunodeficiency [16,17,18,19]. Thus, targeting JAK3 has a more attractive approach for the treatment of immunological diseases. 

In the cells that express JAK3, the function of JAK3 is limited to a γ-chain common to interleukin (IL-2, IL-4, IL-7, IL-9, IL-15, and IL-21) and their receptors [20,21,22,23]. Studies of JAK3-deficient mice and humans showed that JAK3 is a crucial component in cellular signaling that involve immune responses [5,24,25,26]. JAK3 not only affects the development and survival of T cells, but also influences the differentiation of T helper (Th) cells, and is also essential for the differentiation of classic Th2 cells. IL-4 is the most important cytokine controlling Th2 cell differentiation and relies mainly on JAK3 activation for signal transduction. Therefore, the absence of JAK3 will block the differentiation of Th2 cells. At the same time, JAK3 can also influence the differentiation of Th1 cells, but not by direct signaling mechanisms. It is believed that differentiation of Th1 cells is influenced by external modification, and if JAK3 is lacking, the T-bet associated with IFN-γ-promoter activity will be reduced, and the occurrence of Th1 cells producing IFN-γ will also be impaired [27]. In studies of the mechanism of action of IL-21, its role was shown to be mediated through the JAK1/3 STAT3 signaling pathway, especially affecting the differentiation of B cells and Th17 cells. In the case of Th17 cells, the expression of retinoic acid-related orphan receptor gamma t (RORγt) was increased through the JAK3-STAT3 signaling pathway, thus promoting the differentiation of Th17 [28,29]. 

Since the function of JAK3 in cell signaling is usually to promote immune responses, inhibiting JAK3’s function offers a unique possible therapeutic potential for the treatment of patients with abnormal immune responses, including potential application in the treatment of autoimmune diseases. Therefore, a major investigative effort of several research groups is being directed at the study of JAK3 inhibitors. Farmer et al. showed how decernotinib (vx-509), a type of oral JAK3 selective inhibitor, has been evaluated in clinical research on RA. The results of this study showed that JAK3-selective inhibition has significant effectiveness [30]. Since the active site of JAK3 is unique and usually includes cysteine (Cys909), this can be a potentially active target for the development of a JAK3 inhibitor [31]. For example, a recently reported 4-aminopiperidine-based compound RB1 can covalently modify the cysteine residue 909 of JAK3, can inhibit the activity of JAK3, and has no effect on JAK1, JAK2, and TYK2 [32]. Goedken et al. designed a JAK3 inhibitor that can replace the tricycle, and could selectively avoid the inhibition on other JAKs. It displays an inhibitory role mainly through a covalent interaction between electrophile (located in the terminal end of the inhibitor) and an active site cysteine (Cys-909) [33]. Although inhibitors have been reported for JAK3, most of them still remain at the level of animal experimentation. Therefore, more research is needed for evaluation of clinical therapeutic efficacy and identification of potential adverse side effects.

#### 2.1.2. Tyrosine Kinase 2

TYK2, another member of the JAK family, was originally discovered as an important component in the type I IFN-mediated signal way [34]. However, other studies also showed that TYK2 plays a vital role in some immune processes, including the activity of natural killer cells [35] and maturation of B cells [36], and the differentiation of Th1 cells and Th17 cells [37]. In addition, in some autoimmune disease processes, TYK2 abnormal expression is associated with diseases, especially lupus erythematosus [38]. Studies have shown that lack of TYK2 can lead to cell cycle disorders in terminally differentiated B lymphocytes, and lack of TYK2 will increase the levels of immunoglobulin E (IgE) and inhibitor of nuclear factor kappa-B (IκB)-kinase α expression [39]. TYK2 also has an effect on T regulatory (Treg) cells. TYK2-deficient CD4^+^ T cells could not differentiate into induced Treg (iTreg) and lack the expression of Foxp3 [40]. TYK2 also accelerates the CD4^+^ T cell differentiation to Th1 [41] and promotes the development of Th17 [42]. The TYK2/JAK2-STAT3 signaling pathway is a pathway that promotes the differentiation of Th17 cells. Cytokine IL-23 phosphorylates TKY2 and JAK2 through IL-23R, and then activates STAT3, resulting in the up-regulation of RORγt, which promotes the differentiation of Th17 cells and the production of inflammatory factors IL-17 and IL-22 [43,44,45,46]. In a procession of IL-12 promoting Th1 cell differentiation, IL-12 binds to IL-12R, the downstream signal pathway of IL-12R is a TYK2/JAK2-STAT4 signal pathway, and STAT4 is activated and promotes expression of T-bet, resulting in the differentiation of naïve T cells to Th1 and product IFN-γ [47,48]. The TYK2 inhibitor, SAR-20347, was reported to be able to decrease the pathology of psoriasis in mouse models [49]. Additionally, TYK2 can trigger the balance of Th1/Th2 to differentiate cells towards Th1, reducing allergic inflammation by minifying the levels of Th2-mediated IgE and immunoglobulin G (IgG), as well as by decreasing the numbers of eosinophils [41]. A study using TYK2^−^/^−^ mice also observed that TYK2^−^/^−^ mice were less susceptible to collagen-induced arthritis (CIA) and had a lower incidence of arthritis compared with wild type (WT) mice. The macrophage and neutrophil infiltration and articular cartilage fibrillation were all reduced. Moreover, the related cytokines of Th17/Th1, such as IL-6, IL-1β, and matrix metalloproteinase (MMPs) were also reduced in TYK2^−^/^−^ mice [50]. Given TYK2 is widely distributed in the body, it is likely that it has a variety of regulation on different cells. Using TYK2 as a treatment target in autoimmune diseases may have side effects. Therefore, more studies are needed to determine effects and side effects. 

## 3. Transcription Factors

Transcription factors are a group of proteins that combine with promoter regions of specific DNA sequences that lead to transcription and control the expression intensity of gene expression in specific time and space. According to this function, transcription factors can be divided into two categories. The first are a group of general transcription factors that create a transcription initiation complex together with RNA polymerase II to assure that transcription is initiated at the correct position. The second group of transcription factors are found at specific tissue or cellular sites, and are not active until expressed by specific molecular proteins at these tissue or cellular sites or after being stimulated by proteins produced at these sites by translation. In human cells, there are more than 1000 families of DNA-binding transcription factors that regulate gene expression in the form of gene specificity [51].

### 3.1. Forkhead Box Protein 3

Forkhead box protein 3 (Foxp3) is the main transcription factor involved in the differentiation and function of Treg cells, and its specific expression on Treg cells provides an important biomarker of Treg cells [52,53]. Treg cells have been shown to prevent inflammatory and autoimmune diseases in experimental murine models by inhibiting the pathologic response of Th effector cells [54,55,56]. In mouse models of asthma, Foxp3 can activate Treg cells and inhibit NK cells [57]. Transforming growth factor β (TGF-β) is a key factor that induces cells to express Foxp3, mainly through the TGF-β/SMADs (drosophila mothers against decapentaplegic) signaling pathway to activate Foxp3 and promote the differentiation of naïve T cells into Treg cells [58]. Defective or down-regulated Foxp3 has been shown to contribute to the loss of Treg cell function and the concomitant development of autoimmune disease [59]. The functional role of Treg cells is facilitated by their capacity to align themselves in close juxtaposition to their targets. Once at these collocated sites, Tregs of human and murine origin have been shown recently to produce their in vitro and in vivo effects by Foxp3-induced transactivation of chemokine (C-C motif) ligand 3 (CCL3) and CCL4 gene expression, followed by CCL3 and CCL4 chemokine production and attraction of CD4^+^ and CD8^+^ T cells, thus fulfilling a direct regulatory function [59]. Foxp3 can bind to more than 2800 gene loci, of which approximately 700–1400 genes are related to the development of Treg cells [60,61,62].

Foxp3 is typically regulated by phosphorylation, acetylation, and ubiquitination [63]. For example, the Foxp3 serine 422 site can be phosphorylated by Pim-1 kinase, leading to a decline in its functional activity [64]. Pim-1 kinase can, therefore, be considered as a potential target for treatment and prevention of autoimmune disease. Similarly, the Pim-2 kinase has also been shown to phosphorylate and modify Foxp3 at several sites in the amino-terminal area of Foxp3, thereby reducing the inhibitory function of the Treg. Meanwhile, Pim-2 knock out (KO) mice show enhanced suppressive function and lineage stability of Treg cells [65]. The suppressive function of Treg cell can be up-regulated by either pharmacologically inhibiting Pim-2 kinase activity or by genetically knocking out Pim-2 in rodent Treg cells [65]. In addition, Foxp3’s Ser, Thr, and Tyr residues can be phosphorylated by cyclin-dependent kinase 2 (CDK2), causing damage to the function of Foxp3 and Treg inhibitory activity. Concurrently, Morawski et al. [66] provided evidence that removal of the CDK2 motif was able to improve the stability of Foxp3 and Treg function. A novel differentially methylated region (DMR) in the upstream of the Foxp3 promoter was shown to display higher sensitivity to methylation-induced silencing. This new DMR also has relevance to the regulation of Foxp3 [67]. Acetylation also plays an important role in maintaining Foxp3 protein expression levels and transcriptional activity, as well as in Treg cell-mediated suppression. It has been reported that Mammalian sterile 20-like kinase 1 (Mst1) could indirectly regulate the stability of Foxp3 through inhibiting the activity of Sirt 1, a lysine deacetylase which can deacetylate Foxp3, to improve the acetylation of Foxp3. It also found that Mst1 could directly interact with Foxp3 to interfere the interaction between Sirt1 and Foxp3, eventually regulating the function of Treg [68]. Transcription factor Foxp3 can also be regulated by polyubiquitination of multiple lysine residues, deubiquitinase (DUB) USP7 was found to be related to the Foxp3 in the nucleus. Expression of USP7 can reduce Foxp3 polyubiquitination and increase the expression of Foxp3. Consequently, the application of DUB inhibitors or knock out USP7 will reduce endogenous Foxp3 protein and the inhibition function of Treg in vitro [69]. Collectively, these observations suggest an important role of Foxp3 in autoimmunity, and strongly support a rationale for indirectly or directly targeting Foxp3 as a treatment stratagem for autoimmune diseases [70,71].

Currently, many molecules associated with Foxp3 have been reported to regulate the function of Treg by interacting with Foxp3. KRAB-associated protein 1 (KAP1) is the binding partner of Foxp3 in human Treg cells; KAP1-deficient Tregs cannot induce the Foxp3-regulated gene. KAP1 regulates the function of Treg in a way that relies on Foxp3, but its regulation of Treg differentiation does not depend on Foxp3 [72]. Since Helios^+^ Foxp3^+^ CD4^+^ (Helios^+^) Treg cells are involved in the regulation of multiple autoimmune diseases, they can potentially improve the expression of various Treg-related molecules and the function of induced Treg cells. However, Helios in Foxp3-deficient CD4^+^T cells cannot improve the inhibitory function of induced Treg cells, indicating that Helios plays a role that needs to work concomitantly with Foxp3 [73]. In a sepsis model, pharmacological inhibition of both adenosine and c-jun n-terminal kinase (JNK) was shown to reduce Foxp3 protein levels. JNK/activator protein 1 (AP-1) activation and Foxp3 protein expression levels decreased in CD4^+^CD25^+^ Treg cells. This study showed that adenosine plays significant roles in the high expression of Foxp3, and adenosine promotes Foxp3 expression in Treg cells via the JNK/AP-1 pathway [74]. Thus, the Foxp3 transcription factor is considered a dominant regulator for Treg cell development and function is directly regulated by multiple posttranslational modifications that occur in response to various external stimuli. Many molecules have been reported to interact with Foxp3; thus, targeting these molecules directly or indirectly could regulate the function of Treg and improve autoimmune diseases. However, the specificity of these molecules on Foxp3 remains to be further studied.

### 3.2. Retinoic Acid-Related Orphan Receptor Gamma t

The retinoic acid-related orphan receptor γ (RORγ) is a member of the nuclear receptor superfamily, which belongs to the ROR nuclear receptor subfamily [75]. RORγ has two subtypes, RORγ1 and RORγt. These two subtypes differ only in their amino-terminal region and share the same ligand binding domain (LBD). RORγ1 can be expressed in many tissues, but the expression of RORγt is limited to the cells of the immune system, including Th17 cells, γδT cells, LTi cells, and NKT cells. RORγt has been shown to be a type of transcription factor that is controlled by the ligand and thereby controls the expression of the pro-inflammatory factors in several autoimmune diseases. It plays a determinative role primarily by controlling the cytokines that are produced by the immune cells, including IL-17 and the IL-22, that are crucial in the pathogenesis and progression of various immune diseases [76,77,78]. Therefore, RORγt is a potential small molecule therapeutic target for the treatment of many autoimmune diseases.

Th17 cells play an important role in autoimmunity, particularly in relation to many pathological processes of experimental autoimmune encephalomyelitis (EAE) and collagen induced arthritis (CIA). Since Th17 cells can secrete IL-17A, and IL-17A is a pro-inflammatory cytokine that can cause tissue damage, this provides a negative factor in the long-term control of chronic inflammatory and autoimmune diseases [79,80]. The naïve CD4^+^T cell that overexpresses RORγt can be induced toward to Th17, and the differentiation of Th17 in mice with RORγt^−^/^−^ is impaired [75]. Th17 cells can significantly express RORγt, and RORγt could control the function of Th17 cells. RORγt is necessary to induce the transcription of IL-17A, and some autoimmune diseases are dependent on Th17 cells [75]. Several research groups have found that small molecule inhibitors of RORγt can inhibit the function of Th17 cells in vitro and in vivo [78]. This suggests that targeting on RORγt is a feasible way to control autoimmune diseases. Cardiac glycoside digoxin has a specific inhibitory effect on the transcription activity of RORγt; it inhibits the differentiation of Th17 cells in mice and does not affect other T cell lines. It can reduce the severity of autoimmune diseases in mice; however, digoxin is toxic to human cells. Its non-toxic synthetic derivatives, 20,22-dihydrodigoxin-21,23-diol (Dig(DHD)) and digoxin-21-salicylidene, have been shown to inhibit the induction of IL-17 in human CD4^+^T cells [81]. SR1555, a new synthetic specific ligand of RORγt, is reported not only to inhibit the development and function of Th17 cells but also to improve the frequency of Treg cells [82]. SR1001, a kind of high-affinity synthetic ligand, has specificity for RORγt and RORα and could inhibit the differentiation and function of Th17 cells. It inhibits the development of mouse Th17 cells by inhibiting the expression of IL17A gene and the production of protein. When SR1001 is added to the differentiated mouse and human Th17 cells, it can inhibit the expression of cytokines, and SR1001 also proved to be effective in inhibiting the clinical symptoms of autoimmune diseases in mice [83]. Bio-0554019, a selective RORγ inhibitor, can limit the expression of gene dependent on RORγ in the Th17 cells, δT cells, and memory T cells. It also inhibits the reaction of acute IL–17 in vivo [84] (Table 1). Studies have reported that the expression of RORγt is associated with the generation of IL-17, but there is not much of an association with the encephalitogenicity of myelin-specific CD4 T cells. Use of small interfering RNA (si-RNA) inhibits the expression of RORγ on myelin-specific CD4 T cells; however, this does not reduce the severity of adoptively transferred EAE, indicating that in terms of encephalitis CD4 T cells, RORγt is not an effective therapeutic target [85].

### 3.3. T-Box Expressed in T Cells 

T-box expressed in T cells (T-bet) is also known as TBX21. T-bet is expressed in a large variety of immune cells [86] and has a unique effect on the differentiation of three helper T cell populations, promoting Th1 differentiation while suppressing Th2 and Th17 development [87,88,89]. The isolated T-bet can control Th1 gene program in naïve CD4^+^ T cells, T-bet directly activates the gene which encodes IFN-γ, and the abnormal T-bet is able to shift the fully differentiated Th2 cells into Th1 lineage [87]. T-bet may also be associated with the expression of genes that control Th1 cytotoxicity, such as FasL and perforin [4]. If naïve CD4^+^ T cells overexpress T-bet in mice with damaged immune systems, this could cause long-term enteritis regulated by Th1; in contrast, naïve CD4^+^ T cells with defective T-bet are incapable of inducing the disease [90]. During Th1-mediated inflammation, T-bet can control the expression of gene of small molecular regulators that affect the migration of Treg cells in the scurfy mouse model of autoimmunity; in contrast, T-bet-deficient Treg cells cannot control the response of Th1 [91]. Moreover, T-bet has been shown to be antagonistic to the proliferation of Th cells. Although defective T-bet can significantly increase the proliferation of Th cells, it may inhibit cell proliferation in a way that does not depend on IFN-γ and IL-2 [92]. GATA binding protein 3 (GATA-3) is also a transcription factor for Th2, but it can be also expressed in Treg cells; the singular absence of the GATA-3 or T-bet gene on Treg cells is not essential for Treg function. However, a dual absence of these moieties can lead to severe autoimmune diseases. The obliteration of Treg cell function is related to the up-regulation of transcription factor RORγt and the down-regulation of Foxp3; both GATA-3 and T-bet can inhibit the expression of RORγt [93]. These results show that the essential transcription factors in autoimmune responses are interconnected with each other. The expression of T-bet in Treg can be induced independently of IFN-γ, which is necessary to control Th1 autoimmunity, and is strongly associated with the strength of T cell receptor (TCR) signaling [94]. T-bet overexpression in T cells can inhibit the development of autoimmune arthritis, which may be related to the down-regulation of Th17 cell differentiation [95]. Many immune-related diseases, such as asthma and systemic sclerosis, are correlated with T-bet. In different types of autoimmune diseases, T-bet can act differently.

Modulating the expression of T-bet is a potentially feasible stratagem for preventing and treating autoimmune diseases. It has been reported that USP10, a carboxyl-terminal ubiquitin-processing protease, can interact with T-bet in the cell nucleus. The overexpressed USP10 can directly inhibit the ubiquitin of T-bet and increase the expression of T-bet, so a T-bet inhibitor, which has been reported, regards USP10 as a target molecule. USP10 is also able to maintain a high level of expression of T-bet or IFN-γ in inflammation antagonism caused by Th2 cells [96]. Lysine 313 of the T-box area is also critical to controlling the stability of the T-bet protein, and ubiquitin lysine 313 of T-bet can be degraded by protease [97]. Although there are many reports on the correlation between T-bet and development of the disease or immune cellular function, there is a paucity of studies that have evaluated the use of T-bet as a therapeutic target for treatment of the autoimmune diseases.

## 4. Conclusions

The pathogenesis of autoimmune diseases is related to a variety of factors; kinases and transcription factors are only parts of them, but may be crucial in the initiation and development of these diseases. Inhibiting the activity of the JAKs kinase family can reduce the function of immune response cells. In contrast, the manipulation of Foxp3 stability and its activity ensures the function of Treg cells. For example, all-trans retinoic acid (atRA), a vitamin A metabolite, has been shown to promote differentiation and help maintain the expression of Foxp3 in Foxp3^+^ Treg cells [98,99,100]. RORγt is required in Th17-dependent autoimmune disease, and there are many reports about the inhibitors of RORγt. T-bet has different roles in different types of autoimmune diseases, but there is less medical research on it, and the functional mechanism remains to be explored. Because the medical research in the field of kinases and transcription factors still mostly involves experimental animals, there are not enough clinical data. More research is needed to validate their role and determine possible side effects in the human.

## Figures and Tables

**Table 1:** Associated inhibitors.

Inhibitors	Target	Function
Decernotinib (vx-509)	JAK3	Inhibit JAK3
4-aminopiperidine-based compound RB1	JAK3	Inhibit JAK3
Tricyclic Jak3 inhibitor	JAK3	Inhibit JAK3
SAR-2034	JAK1 and TYK2	Inhibit JAK1 and TYK2
Cardiac glycoside digoxin	RORγt	Inhibit Th17 *cell differentiation*
SR1555	RORγt	Inhibit Th17 cell development and increase the frequency of Treg
SR1001	RORγt and RORα	Inhibit IL-17A gene expression and protein production
Bio-0554019	RORγt	Inhibit Th17 differentiation and RORγ signature gene expression
